# Polymer encapsulation of anticancer silver–N-heterocyclic carbene complexes†

**DOI:** 10.1039/c8ra00450a

**Published:** 2018-03-15

**Authors:** H. A. Mohamed, M. Khuphe, S. J. Boardman, S. Shepherd, R. M. Phillips, P. D. Thornton, C. E. Willans

**Affiliations:** School of Chemistry, University of Leeds Woodhouse Lane Leeds LS2 9JT UK c.e.willans@leeds.ac.uk; Department of Pharmacy, University of Huddersfield Queensgate Huddersfield HD1 3DH UK

## Abstract

Amphiphilic block copolymers have been developed for the encapsulation of organometallic drugs. silver–N-heterocyclic carbene complexes have shown significant promise as anticancer and antibacterial compounds, and have been studied as the payload in these carriers. Simple modification of the N-heterocyclic carbene ligand structure enables solubility properties and interaction with the polymer to be tuned.

## Abstract 2

Amphiphilic block copolymers have been developed for the encapsulation of silver anticancer drugs.

## Graphical abstract

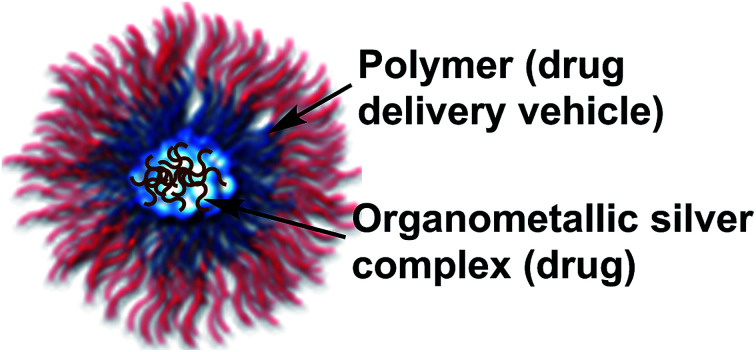

## Introduction

Silver has long been established as having antibacterial properties,^1^ and more recently the chemotherapeutic effects of silver have been recognised.^2,3^ Silver salts, nanoparticles and compounds such as silver sulfadiazine have been used in healthcare. Recently, silver–N-heterocyclic carbene (NHC) complexes have shown significant promise as potential antibacterial and anticancer drugs,^4–9^ and are thought to have a relatively slow silver release rate which is ideal for prolonged activity. The *in vitro* effects of silver–NHCs, however, do not usually translate into comparable *in vivo* activity, due to lack of targeting and change in metal speciation upon entering a biological environment.^10^ It is therefore desirable to develop delivery systems which act to protect the drug, target the site of action and release the drug in response to specific stimuli.

Polymer-based drug delivery systems have demonstrated extensive promise for the controlled release of organic therapeutics at a target site *in vivo*.^11^ The polymer acts to prevent the premature metabolism of the drug prior to its intended deployment,^12^ and also protects healthy cells from exposure to what are often cytotoxic drug molecules.^13^ Enhanced drug concentrations are consequently found at the diseased cells, enabling lower concentrations to be administered and ensuring that the side-effects that are associated with treatments, such as chemotherapy, are restricted.^14^ Although extensive research has been undertaken to develop polymeric systems for the controlled delivery of organic drugs, the controlled release of organometallic payloads as drug delivery systems is relatively under-developed.^15^ Cisplatin has been incorporated into polymeric micelles,^16–18^ and Youngs and co-workers have reported drug-delivery vehicles for silver–NHC complexes, in the form of polymer-based nanoparticles,^19^ with the expansion of this highly-promising research domain being of great medicinal value. Herein, we report organometallic drug encapsulation within biodegradable polymeric nanoparticles, and an assessment of the cytotoxic profile of the nanoparticles produced. Significantly, a novel loading approach in which the silver–NHC complex mediates transformation of water-soluble polymers into silver-containing nanoparticles is described and demonstrated. This is possible as many of the polymers used in this study contain functional groups capable of ionic crosslinking to yield nanoparticles. Such functional groups also permit nanoparticle modification with cell-binding groups, an essential feature for the future development of targeted drug delivery systems.

## Results and discussion

**Fig. 1:**
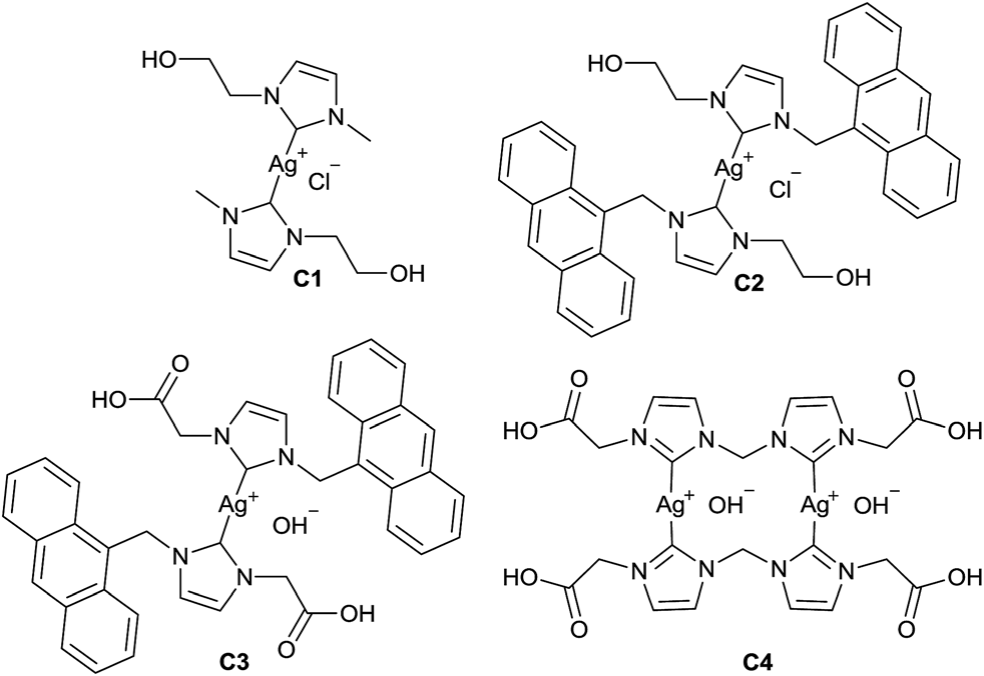
Cytotoxic silver–NHC complexes studied for encapsulation within polymeric nanoparticles.

Silver–NHC complexes used in this study (Fig. 1) were chosen based upon their aqueous solubility (C1, C2), fluorescent properties (C2, C3), and the ability to form electrostatic interactions with cationic polymers, through interaction with anionic carboxylate groups (C3, C4).

**Fig. 2:**
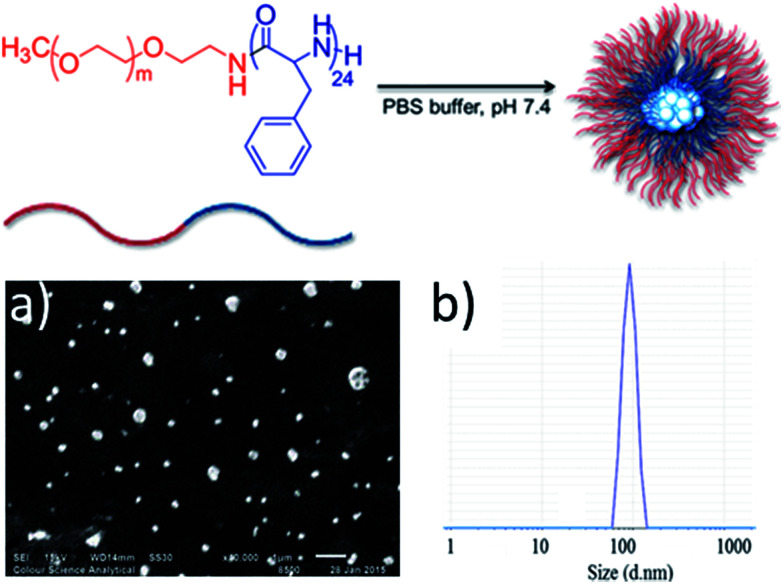
The envisaged self-aggregation of P1 into particles that can physically entrap silver(i)–NHC complexes (top). (a) SEM microphotograph showing the formation of nanoparticles, scale bar represents 1 μm. (b) DLS data revealing the size distribution of particles.

Initially, methoxy-poly(ethylene glycol)-*b*-poly(phenylalanine) (P1) (synthesised as mPEG_113_-*b*-poly(Phe)_24_) was used to determine the feasibility of encapsulating organometallic compounds within poly(amino acid)-containing polymers. P1 is an amphiphilic co-polymer that can self-assemble in aqueous media (Fig. 2), and due to the phenylalanine content is susceptible to hydrolysis by chymotrypsin, a digestive enzyme that is secreted by the pancreas.^12^ Self-assembly of P1 was demonstrated through its addition to water (10 μg mL^−1^). Scanning electron microscopy (SEM) confirmed the production of nanoparticles (Fig. 2a) that possessed an average size of 121 nm, as determined by dynamic light scattering (DLS) (Fig. 2b).

**Fig. 3:**
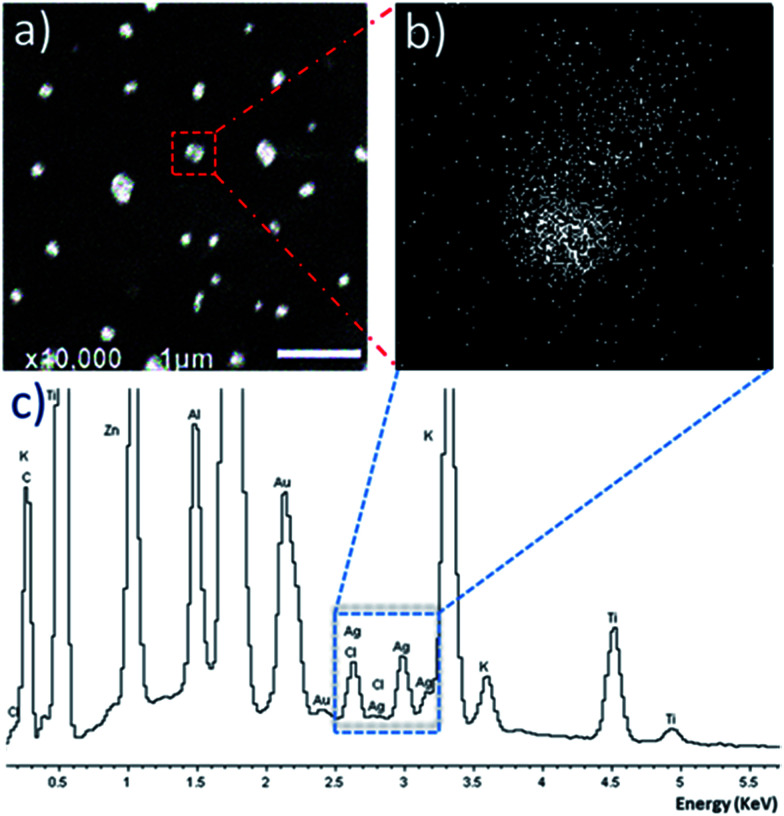
(a) SEM image of silver–NHC C1 loaded P1 particles. (b) EDX map corresponding to an area that is located within a loaded particle. (c) EDX spectrum corresponding to an area that is located within the loaded particle, showing the presence of silver. Other elements that appear on the spectrum emanated from the composition of the SEM glass cover slide and from the gold-coating.

Encapsulation of a silver–NHC by P1 was investigated using the water-soluble complex C1, with varying ratios of P1 : C1 (ESI†). DLS analysis revealed an increased average particle size of 151.7 nm and PDI of 0.307 when using 0.8 : 1 ratio of P1 : C1. SEM analysis of P1 : C1 revealed the formation of discrete particles (Fig. 3a), with energy-dispersive X-ray spectroscopy (EDX) mapping the location of silver ions. SEM-EDX reveals that the silver ions are located within the particle region, indicating successful encapsulation of silver–NHC C1 (Fig. 3b). The EDX spectrum shows peaks corresponding to silver and chloride ions, supporting the presence of the complex within the particle region (Fig. 3c).

**Fig. 4:**
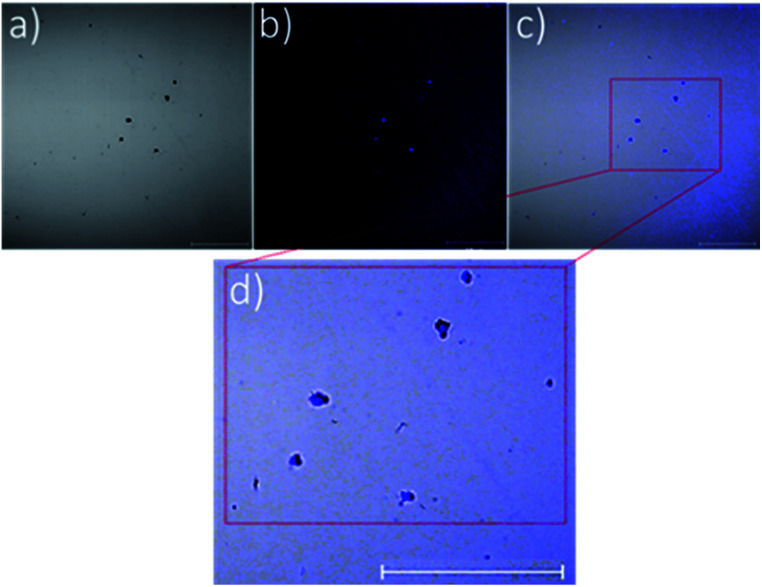
Confocal microscopy images obtained from C2-loaded mPEG_5000_-*b*-poly(Phe)_24_ particles: (a) under visible light, (b) under fluorescence, (c) an overlay of the two images, (d) highly resolved image revealing that the blue fluorescent spots (*i.e.* assumed to be the molecules of the encapsulated complexes) occupy the same positions as the black clusters (which are assumed to be the polymeric shell). Scale bars represent 1 μm.

To emphasise further the encapsulation of silver–NHCs in P1 particles, the intrinsic fluorescence property of complex C2 (Fig. 1) was exploited to map the location of the complex in the particles. Confocal laser scanning microscopy was used to image the C2-loaded particles. Imaging was carried out under visible light to map the polymeric shell (Fig. 4a) and under fluorescence to map the inherently fluorescent silver–NHC complex (Fig. 4b). These images were overlaid (Fig. 4c) and resolved further to reveal clearly the two distinct regions in the particles (Fig. 4d). The discrete clusters that fluoresced, and were thus assumed to be the silver–NHC complex, occupy positions that coincide with the positions of the dark spots (assumed to be the discrete polymeric particles) (Fig. 4c and d). As such, it can be summarised that the silver–NHC complexes either become adsorbed onto the polymeric particles or are encapsulated within the polymer during nanoparticle assembly. The latter possibility is more plausible because of the intrinsic hydrophobicity of the metal complex.

**Fig. 5:**
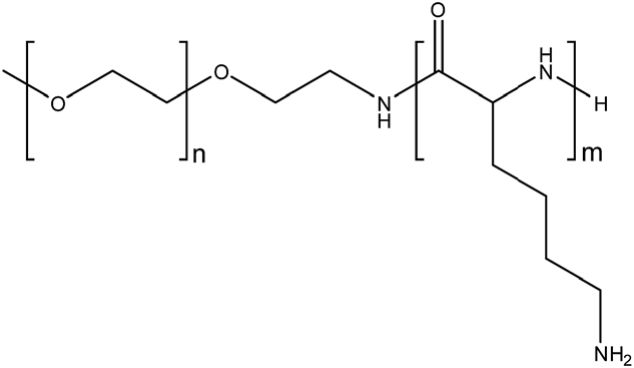
Methoxy-poly(ethylene glycol)-*b*-poly(l-lysine). P2: *n* = 113, *m* = 39. P3: *n* = 113, *m* = 50. P4: *n* = 113, *m* = 140.

The use of organometallic-polymer conjugation to instigate nanoparticle formation was investigated using NHCs with carboxylic acid *N*-substituents (C3 and C4) and amine-presenting polymers, with the aim of forming electrostatic interactions between the deprotonated anionic carboxylate groups and the cationic polymer. Poly(l-lysine) may be employed as a biodegradable, cationic polymer capable of binding with anionic carboxylate groups, hence methoxy-poly(ethylene glycol)-*b*-poly(l-lysine) was used in this study (Fig. 5). This method of encapsulation ensures that nanoparticles are only formed from the water-soluble polymers when contact between the drug and the poly(l-lysine) component of the block copolymer occurs, thereby eliminating the possibility of conjugation of the drug to the surface of pre-formed PNs. Polymers with varying mPEG:poly(l-lysine) ratios (P2, P3 and P4), were prepared using PEG as the macroinitiator for the ring-opening polymerisation of l-lysine *N*-carboxyanhydrides (ESI†).^20^

**Fig. 6:**
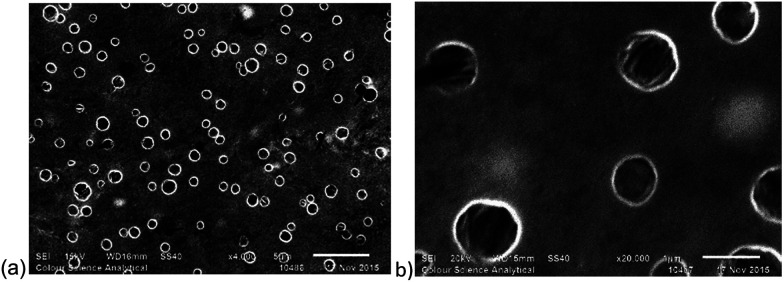
SEM images of P3 : C4. Scale bar represents (a) 5 μM, and (b) 1 μM.

Solutions of the three polymers P2, P3 and P4, and solutions of silver–NHC complexes C3 and C4 were prepared (1 mg mL^−1^). In addition to complexes C3 and C4, the corresponding imidazolium ligand precursors L3 and L4 were also examined. All solutions were initially filtered through PTFE syringes with pore size of 0.45 μm followed by filtration using a filter with a pore size of 0.2 μm to remove all particles that possess a diameter greater than 0.2 μm, as confirmed by DLS analysis. Filtered solutions of each polymer and either silver–NHC complex C3 or C4, or imidazolium L3 or L4, were combined (1 : 1) at 37 °C and analysed using DLS (Table 1). DLS confirmed the absence of particles in the individual polymer, metal complex and imidazolium solutions, by revealing blank DLS charts. When the respective polymer solutions were mixed with silver–NHC complex solutions (1 : 1 v/v), DLS confirmed the formation of particles, by revealing the emergence of size distribution traces that were initially not present in either the independent polymer solutions or in the complex solutions. The particle diameters generally increased with increasing polymer length, as expected. However, in the case of P3 : C4, a larger than expected particle diameter of 452.7 nm was observed which suggests vesicle formation (Fig. 6). Both imidazolium salts and corresponding silver–NHC complexes form particles of similar size, indicating that the ligand plays the major role in particle formation, which is not affected by the presence of silver.

**Table 1:** Polydispersity index (PDI) and average particle size (APS) for conjugated PNs

Polymer	Imidazolium or silver–NHC	PDI	APS (nm)
P2	L3	1.000	146.0
P3	L3	0.754	173.1
P4	L3	0.292	224.2
P2	C3	0.188	159.7
P3	C3	0.173	175.6
P4	C3	0.153	176.5
P2	L4	1.000	121.8
P3	L4	a	a
P4	L4	0.484	253.2
P2	C4	0.249	160.8
P3	C4	0.173	452.7
P4	C4	0.449	221.8

a A reading could not obtained.

Encapsulation of a drug carries the risk of impeding cytotoxic behaviour. To assess the viability of encapsulated silver–NHCs against cancer cells, activities of P1 : C1, P3 : C3 and P3 : C4 were evaluated against the pancreatic adenocarcinoma cell line Panc 10.05 using MTT-based assays. Silver–NHC complexes C1, C3 and C4 were also tested as comparators (Table 2). There is clear evidence from the results presented in Table 2 that encapsulation of silver–NHCs by the polymers investigated in this study does not adversely affect the cytotoxicity of the complexes to Panc 10.03 cancer cells. Selectivity of C1 and C3 does decrease upon encapsulation when the IC_50_ values against non-cancer ARPE-19 cells are compared to IC_50_ values for Panc 10.03, though the selectivity of C4 improves upon encapsulation. These results present significant promise, as encapsulation may feasibly prevent decomposition of the drug before it reaches the intended target site, and may enable an enhanced, and thus more effective concentration of inorganic therapeutic to be delivered to the target site.

**Table 2:** Response of Panc 10.05 and ARPE-19 cells to silver–NHC complexes C1, C3 and C4, and polymeric nanoparticles PN2, PN3B and PN4B. Values presented are IC_50_ μM ± SD for three independent experiments. The selectivity index is defined as the ratio of IC_50_ in non-cancer ARPE-19 cells to the IC_50_ in cancer Panc 10.05 cells

Drug	Panc 10.05	ARPE-19	Selectivity index
C1	30.57 ± 13.16	>100	>3.27
P1 : C1	25.8 ± 4.3	22.70 ± 2.70	1.43
C3	12.3 ± 3.2	>100	>8.15
P3 : C3	8.2 ± 1.8	23.59 ± 6.88	2.86
C4	26.3 ± 11.5	68.70 ± 7.16	2.61
P3 : C4	16.0 ± 5.5	83.37 ± 17.06	3.23

## Conclusions

In conclusion, we have presented novel methods towards organometallic drug delivery systems involving encapsulation within polymeric nanoparticles. The polymers used in this study offer the potential for structural modification, enabling targeted carriers to be developed. The structural diversity of NHC ligands allows the interaction between the polymer and drug to be altered, thus changing the physical properties of the nanoparticles. Importantly, the silver–NHCs studied in this work retain their cytotoxic profile against cancerous Panc 10.05 cells upon encapsulation within the polymers, making these systems viable for organometallic drug delivery. Work is ongoing to understand the drug loading efficiencies and pharmacokinetic behaviour of the drug-loaded nanoparticles, which will enable optimisation of these systems through ligand and/or polymer modification.

## Conflicts of interest

There are no conflicts to declare.

## Supplementary Material

RA-008-C8RA00450A-s001
