# Supplementary material for: Polymer encapsulation of anticancer silver–N-heterocyclic carbene complexes
Source: RSC Adv. 2018 Mar 15;8(19):10474–7. doi: 10.1039/c8ra00450a (PMC9078921; doi:10.1039/c8ra00450a)
Supplement: RA-008-C8RA00450A-s001 [file RA-008-C8RA00450A-s001.pdf]

Electronic Supplementary Material (ESI) for RSC Advances.
This journal is © The Royal Society of Chemistry 2018

Supporting Information

Polymer encapsulation of anticancer silver-*N*-heterocyclic carbene complexes

H. A. Mohamed, M. Khuphe, S. J. Boardman, S. Shepherd, R. M. Phillips, P. D. Thornton and C. E. Willans

Contents

General 2
Synthesis of C1 3
Synthesis of C2 4
Synthesis of C3 4
Synthesis of C4 5
Synthesis of Phenylalanine (Phe) NCA 6
Synthesis of Nε-Carbobenzyloxy-L-Lysine (Lys(Cbz)) NCA 7
Synthesis of P1 7
Synthesis of Cbz-protected P2-P4 8
Deprotection of Cbz-protected P2-P4 8
Self-Assembly of P1 in the presence of C1 10
Creation of nanoparticles by silver-NHC complex-mediated polymer self-assembly 12
Cell line testing 13

General

All chemicals were purchased from Sigma-Aldrich, Alfa Aesar or Thermo Fisher Scientific and used as supplied.

^{1}H and $^{13}C\{^{1}H\}$ NMR spectra were recorded on either a Bruker DPX300 spectrometer (operating frequency 300.1 MHz for ^{1}H and 75.48 MHz for $^{13}C\{^{1}H\}$), a Bruker Avance 500 spectrometer or a Bruker DRX500 spectrometer (both with an operating frequency of 500.13 MHz for ^{1}H and 125.80 MHz for $^{13}C\{^{1}H\}$). All spectra were recorded at 298K in deuterated solvent. Chemical shift values are quoted in parts per million (ppm, δ), coupling constants, J, are quoted in Hertz (Hz) and assignment of $^{13}C\{^{1}H\}$ NMR spectra was aided by $^{13}C\{^{1}H\}$ DEPT135 experiments when necessary.

High-resolution mass spectra were collected on a Bruker Daltonics (micro TOF) instrument operating in the positive ion electrospray mode. Samples were injected directly from feed solutions and acquired over the range *m/z* 50 – 4000. All spectra were recorded using an acetonitrile/water mix as the eluent and a sodium formate solution as a calibrant.

Microanalyses were performed in the School of Chemistry, University of Leeds by Ms. Tanya Marinko-Covell using a Carlo Erba Elemental Analyser MOD 1106 spectrometer.

FTIR spectra were recorded using a Spectrum One spectrophotometer (PerkinElmer) fitted with diffuse reflectance probe with zinc-selenide window. IR spectra were recorded using a globular light source through KBr beamsplitter for the range 4000-400 cm^{-1} using DLATGS detector with KBr window. 32 scans were recorded for each averaged spectrum with a new background recorded after each sample. IR spectra were analysed using the spectroscopy software package OPUS (v 6.5, Bruker Optiks GmbH).

Scanning Electron Microscopy (SEM) experiments were performed using a Pasteur pipette to extract a drop of the solution and deposit it on to an SEM glass cover slip. The sample was allowed to dry in a fume cupboard, after which the cover slip was mounted on an SEM stub using conductive tape. The SEM stub containing the dried sample was sputter-coated with a thin layer of gold using a current of 20 mA for 2 minutes, in a quorum Q150RS sputter-coater. The coated samples were analysed

for particle size and morphology using a JOEL JSM- 6610LV microscope (Oxford Instruments) equipped with a field emission electron gun as an electron source, using a working distance of 11 mm. Accelerated voltage was applied between 5 and 15 kV.

Fluorescence imaging of was performed using a confocal microscope Zeiss LSM88o inverted with Airyscan microscope that consists of Axio Observer Z1, Nano Focusing Piezo Stage and Zlmulti S1 incubator box. The images were taken at 63 x objective. Lasers include diode 405nm, Argon 458, 488, 514 nm, DPSS 561 nm and HeNe 633 nm. Excitation was performed with 405 nm laser and detection was in the range 370-500 nm.

Synthesis of C1

Ligand precursor **L1**: 1-Methyl imidazole (1.84 mL, 24.4 mmol) and 2-chloroethanol (1.63 mL, 24.4 mmol) were heated at 100 °C in a sealed ampoule for 72 hours. Et_2O (30 mL) was added to the mixture and precipitation of the product as a brown oil was aided by sonication. The Et_2O was decanted from the oil and the product dried *in vacuo*. Yield: 3.0 g, 18.5 mmol, 76 %. ^1H NMR (300 MHz, D_2O): δ 8.81 (s, 1H, NC*H*N), 7.57 (s, 1H, NC*H*), 7.51 (s, 1H, NC*H*), 4.37 (t, J = 5.0 Hz, 2H, CH_2), 3.98 (t, J = 5.0 Hz, 2H, CH_2), 3.96 (s, 3H, CH_3). $^{13}C\{^1H\}$ NMR (75 MHz, D_2O): δ 136.2 (N*C*HN), 123.6 (N*C*), 122.1 (N*C*), 59.8 ($\mathit{C}H_2$), 51.6 ($\mathit{C}H_2$), 35.9 ($\mathit{C}H_3$). HRMS (ESI+): Calcd for $C_6H_{11}N_2O$ $[M - Cl]^+$: 127.0871. Found: 127.0866.

Complex **C1**: **L1** (0.8 g, 4.9 mmol) was dissolved in MeOH (30 mL) and silver oxide (0.87 g, 3.8 mmol) was added. The mixture was heated at reflux for 8 hours. The mixture was filtered through celite, and the solvent was removed from the filtrate *in vacuo* to yield a brown solid. Recrystallisation from MeOH/Et_2O furnished the product as a pale yellow solid. Yield: 0.42 g, 1.06 mmol, 22 %. ^1H NMR (300 MHz, DMSO-d_6): δ 7.40 (s, 1H, NC*H*), 7.36 (s, 1H, NC*H*), 4.07 (t, J = 6.0 Hz, 2H, $C\mathit{H}_2$), 3.66 (t, J = 6.0 Hz, 2H, $C\mathit{H}_2$), 3.13 (s, 3H, $C\mathit{H}_3$). $^{13}C\{^1H\}$ NMR (125 MHz, DMSO-d_6): δ 122.0 (N*C*), 121.8 (N*C*), 61.5 ($\mathit{C}H_2$), 54.0 ($\mathit{C}H_2$), 37.8 ($\mathit{C}H_3$). Anal. Calcd for $C_{12}H_{20}AgClN_4O_2$: C, 36.43; H, 5.10; N, 14.16. Found: C, 36.80; H, 5.30; N, 13.80.

Synthesis of C2

Ligand precursor **L2**: 9-(Chloromethyl) anthracene (0.5 g, 2.2 mmol) was dissolved in MeCN (30 mL) and transferred to an ampoule. 1-(2-Hydroxyethyl) imidazole (0.21 mL, 2.2 mmol) was added. The mixture was heated at 90 °C for 24 hours. Excess Et_2O (200 mL) was added to the orange solution to yield the product as an orange solid. The Et_2O was decanted and the solid was dried *in vacuo*. Yield: 0.71 g, 2.1 mmol, 95%. ^{1}H NMR (300 MHz, $CDCl_3$): δ 10.23 (s, 1H, NC*H*N), 8.61 (s, 1H, Ar), 8.39 (d, 2H, J = 5.3 Hz, Ar), 8.08 (d, 2H, J = 5.3 Hz, Ar), 7.66 (t, 2H, J = 4.7 Hz, Ar), 7.53 (t, 2H, J = 4.7 Hz, Ar), 7.14 (s, 1H, NC*H*), 6.77 (s, 1H, NC*H*), 6.55 (s, 2H, CH_2), 4.41 (t, 2H, J = 5.6 Hz, CH_2), 4.04 (t, 2H, J = 5.6 Hz, CH_2). $^{13}C\{^1H\}$ NMR (75 MHz, $CDCl_3$): δ 137.5 (*C*), 131.4 (*C*), 131.1 (*C*), 130.7 (*C*H), 129.5 (*C*H), 128.5 (*C*H), 125.7 (*C*H), 122.9 (*C*H), 122.0 (*C*H), 121.0 (*C*H), 59.9 (*C*H_2), 52.9 (*C*H_2), 46.0 (*C*H_2). HRMS (ESI+): Calcd for $C_{20}H_{19}N_2O$ $[M - Cl]^+$: 303.1492. Found: 303.1483. IR v_{max}/cm^{-1}: 3233 (OH), 1548-1402 (CC arom). Anal. Calcd for $C_{20}H_{19}ClN_2O·4/3\ H_2O·1/3$ MeCN: C, 65.92; H, 6.07; N, 8.68. Found: C, 66.25; H, 5.90; N, 8.40.

Complex **C2**: **L2** (0.3 g, 0.89 mmol) and silver oxide (0.12 g, 0.53 mmol) were added to a Schlenk flask with activated 4Å molecular sieves and degassed. Anhydrous MeCN (20 mL) and MeOH (14 mL) were transferred to the Schlenk flask, and the mixture was stirred at room temperature for 18 hours. The solution was filtered through celite and the solvents removed *in vacuo* rendering the product as a light yellow solid. Yield: 0.24 g, 0.32 mmol, 36%. ^{1}H NMR (300 MHz, $CDCl_3$): δ 8.53 (s, 1H, Ar), 8.32 (d, 2H, J = 8.4 Hz, Ar), 8.05 (d, 2H, J = 8.4 Hz, Ar), 7.54-7.44 (m, 4H, Ar), 6.89 (s, 1H, NC*H*), 6.50 (s, 1H, NC*H*), 6.24 (s, 2H, CH_2), 4.33 (m, 2H, CH_2), 3.94 (m, 2H, CH_2). $^{13}C\{^1H\}$ NMR (75 MHz, $CDCl_3$): δ 134.1 (*C*), 131.4 (*C*), 131.0 (*C*), 129.7 (*C*H), 129.5 (*C*H), 129.5 (*C*H), 127.6 (*C*H), 122.9 (*C*H), 125.5 (*C*H), 123.3 (*C*H), 121.7(*C*H), 61.6 (*C*H_2), 55.0 (*C*H_2), 47.6 (*C*H_2). HRMS (ESI+): Calcd for $C_{40}H_{36}AgN_4O_2$ $[M - Cl]^+$: 711.1884. Found: 711.1879. Anal. Calcd for $C_{40}H_{36}AgClN_4O_2·2H_2O$: C, 61.27; H, 5.14; N, 7.15. Found: C, 60.90; H, 5.00; N, 7.50.

Synthesis of C3

Ligand precursor **L3**: 1-(Methylanthracene)imidazole (0.15 g, 0.58 mmol) and chloroacetic acid (0.06 g, 0.58 mmol) were dissolved in MeCN (30 mL) and the solution was heated at 90 °C for 24 hours in a sealed ampoule. A yellow solid

precipitated out of solution after 1 hour of heating. The mixture was filtered, and the yellow solid was washed with Et_2O (90 mL) and dried *in vacuo*. The product was recrystallised from CH_2Cl_2/Et_2O and MeCN/Et_2O, and isolated as a hygroscopic yellow solid. Yield: 0.09 g, 0.3 mmol, 46 %. ^{1}H NMR (300 MHz, $CDCl_3$): δ 11.30 (s, 1H, NC*H*N), 8.62 (s, 1H, Ar), 8.31 (d, J = 8.9 Hz, 2H, Ar), 8.08 (d, J = 8.9 Hz, 2H, Ar), 7.69 (t, J = 6.6 Hz, 2H, Ar), 7.69 (t, J = 6.6 Hz, 2H, Ar), 6.60 (s, 2H, C*H*$_2$), 5.23 (s, 2H, C*H*$_2$). ^{13}C{^{1}H} NMR (75 MHz, DMSO-d_6): δ 129.9, 129.1, 129.0, 127.5, 127.0, 125.3, 125.1, 123.7, 123.4, 123.1, 122.3, 121.9, 49.9 (*C*H$_2$), 44.8(*C*H$_2$). HRMS (ESI+): Calcd for $C_{20}H_{17}N_2O_2$ [M - Cl]$^+$: 317.1290. Found: 317.1288. IR v_{max}/cm^{-1}: 3049, 2833 (carboxylic acid OH), 1711 (carboxylic acid C=O), 1256, 1156. Anal. Calcd for $C_{20}H_{17}ClN_2O_2{\cdot}CH_2Cl_2{\cdot}CH_3CN{\cdot}1/3H_2O$: C, 56.98; H, 4.71; N, 8.67. Found: C, 56.90; H, 4.80; N, 8.70.

Complex **C3**: **L3** (0.05 g, 0.14 mmol) and silver oxide (0.42 g, 1.81 mmol) were added to an ampoule with 4Å activated molecular sieves, and dried *in vacuo*. Anhydrous CH_2Cl_2 (7 mL) and MeOH (7 mL) were transferred to the ampoule and the mixture was stirred at room temperature for 24 hours. The solution, which had turned an orange colour, was filtered through celite, and the solvents were removed from the filtrate *in vacuo*. The red/orange hygroscopic solid was recrystallised from MeCN/Et_2O. Yield: 0.02 g, 0.024 mmol, 18 %. ^{1}H NMR (300 MHz, MeOD): δ 8.34 (m, 4H, Ar), 8.20 (m, 4H, Ar), 7.77-7.25 (m, 10H, Ar), 6.46 (broad s, 4H, C*H*$_2$), 6.11 (broad s, 4H, C*H*$_2$). ^{13}C{^{1}H} NMR (75 MHz, MeOD): δ 132.1,131.5, 130.3,130.1, 128.8, 128.3, 126.1, 126.0 (Ar), 123.5, 122.7 (N*C*), 69.4 (*C*H$_2$), 30.9 (*C*H$_2$). Anal. Calcd for $C_{40}H_{32}AgClN_4O_4{\cdot}3/2CH_3CN{\cdot}3/2CH_2Cl_2$: C, 55.27; H, 4.33; N, 7.97. Found: C, 55.50; H, 4.40; N, 8.35.

Synthesis of C4

Ligand precursor **L4**: 1.1'-Methylene-bis-1H-imidazole (0.3 g, 2.0 mmol) was dissolved in MeCN (5 mL) and ^{t}butyl-chloroacetate (0.58 mL, 4.0 mmol) was added. The mixture was heated at 90 °C in a sealed ampoule for 72 hours, and Et_2O (80 mL) was added resulting in a pale yellow solid. The Et_2O was decanted and the solid was recrystallised from MeCN/Et_2O to give a white hygroscopic solid. Yield: 0.6 g, 1.9 mmol, 93%. ^{1}H NMR (300 MHz, CD_3CN): δ 10.65 (s, 2H, NC*H*N), 8.72 (s, 2H, NC*H*), 7.51 (s, 2H, NC*H*), 7.39 (s, 2H, bridging-C*H*$_2$), 5.02 (s, 4H, C*H*$_2$), 1.48 (s, 18H,

CH_3). $^{13}C\{^1H\}$ NMR (125 MHz, CD_3CN): δ 165.9 (*C*=O), 140.5 (N*C*HN), 125.0 (N*C*), 123.6 (N*C*), 84.9 (*C*(CH_3)$_3$), 58.4 (bridging-CH_2), 51.9 (CH_2), 28.2 (C(CH_3)$_3$). IR $ν_{max}$/cm^{-1}: 3080, 2977, 2930, (carboxylic acid OH), 1740 (carboxylic acid C=O), 1153, 749, 615.

Complex **C4**: **L4** (0.2 g, 0.42 mmol) and silver oxide (0.19 g, 0.84 mmol) were added to activated molecular sieves 4Å in a Schlenk flask and dried *in vacuo*. Anhydrous MeCN (10 mL) and anhydrous MeOH (10 mL) were transferred to the Schlenk flask and the mixture was stirred at room temperature for 24 hours. The solution, which had turned grey, was filtered through celite, and the solvent removed from the filtrate *in vacuo* to yield the product as a white solid. Yield: 0.15 g, 0.2 mmol, 46 %. ^1H NMR (300 MHz, CD_3OD): δ 7.60 (s, 4H, NC*H*), 7.33 (s, 4H, NC*H*), 7.24 (s, 4H, bridging-CH_2), 6.48 (broad s, 4H, OH), 4.60 (s, 8H, CH_2). $^{13}C\{^1H\}$ NMR (125 MHz, CD_3OD): δ 124.1 (*C*=O), 121.3 (N*C*),120.4 (N*C*), 55.5 (CH_2), 51.8 (CH_2). IR $ν_{max}$/cm^{-1}: 3366 (OH), 3099, 2966 (carboxylic acid OH), 1742 (carboxylic acid C=O), 1368, 1170, 727. Anal. Calcd for $C_{22}H_{26}Ag_2N_8O_{10}·CH_3OH$: C, 34.09; H, 3.73; N, 13.83. Found: C, 34.20; H, 3.90; N, 13.50.

Synthesis of Phenylalanine (Phe) NCA

Phe (5.0 g, 30.2 mmol) was weighed into a pre-dried three-neck round bottom flask that was evacuated and nitrogen purged three times prior to being used. Anhydrous EtOAc (60 mL) and α-pinene were added (8.23 g, 60.4 mmol) and the resultant suspension heated to reflux. Triphosgene (11.87 g, 40.0 mmol) was dissolved in anhydrous EtOAc (20 mL) and the solution added dropwise *via* a dropping funnel into the refluxing reaction, over 30 minutes. The reaction was heated at reflux for a further 5 hours, after which time the initial suspension had turned into a solution. The volume was reduced to a third of its initial volume using rotary evaporation. The concentrated solution was added dropwise to ice-cold hexane (200 mL) and subsequently stored at -18 °C for 12 hours to allow the NCA to precipitate fully out of solution. The crude NCA was isolated by filtration under vacuum and purified by repeated recrystallisation from EtOAc:n-hexane (1:6 v/v), yielding the pure NCA as cream platelet crystals. The NCA was subsequently stored at -18 ˚C and shielded from moisture. Yield: 4.33 g, 22.7 mmol, 74.9%. ^1H NMR (500 MHz, DMSO-d_6): δ 9.08 (s, 1H, N*H*), 7.33 - 7.17 (m, 5H, Ar), 4.79 - 4.77 (t, 1H, αC*H*, *J* = 10 Hz), 3.03 -

3.02 (d, J = 5 Hz, 2H, CH_2). $^{13}C\{^1H\}$ NMR (125 MHz, DMSO-d_6): δ 172.9 (CH*C*(O)O), 154.7 (CHNH*C*OO), 137.6 (Ar), 129.4 (Ar), 129.1 (Ar), 127.2 (Ar), 56.66 (α*C*H), 38.32 (Ar-*C*H_2). FTIR: ν_{max}/cm^{-1} (solid): 3247 (NH, amide), 2922 (CH, alkyl), 1860, 1778 (CO, anhydride), 859 (C-H, Ar).

Synthesis of Nε-Carbobenzyloxy-L-Lysine (Lys(Cbz)) NCA

The NCA of Lys(Cbz) was synthesised in anhydrous EtOAc, following an analogous procedure to the one described for the synthesis of Phe NCA. Yield: 4.83 g, 15.8 mmol, 88.1% (White crystals). ^{1}H-NMR (500 MHz, DMSO): δ 9.09 (s, 1H, N*H*), 7.39 - 7.26 (m, J = 65 Hz, 5H, Ph), 7.25 (s, 1H, COON*H*), 5.06 (s, 2H, Ar-CH_2), 4.45 - 4.43 (t, J = 10 Hz, 1H, CH), 3.02 - 2.98 (q, J = 20 Hz, 2H, CH_2), 1.78 - 1.63 (m, J = 75 Hz, 2H, CH_2), 1.46 - 1.26 (m, J = 100 Hz, 4H, CH_2CH_2). $^{13}C\{^1H\}$ NMR (125 MHz, DMSO-d_6): δ 172.4 (CH*C*(O)O), 157.8 (CH_2NH*C*OO), 154.1 (*C*HNHCOO), 137.0, (Ar), 128.5 - 128.07 (Ar), 67.6 (Ar- *C*H_2), 54.4 (α*CH*), 41.5 (NH*C*H_2), 29.8 (NHCH_2CH_2), 29.3 (CHCH_2), 24.2 (CHCH_2CH_2). Melting Point: 100 °C. ESI-MS: Calcd for $C_{15}H_{18}N_2NaO_5$ [M+Na]$^+$: 329.111. Found: 329.111. FTIR: ν_{max}/cm^{-1} (solid): 3341 (NH), 2929 (CH), 1856, 1800 (CO anhydride), 1774 (benzyl ester), 1683 (Ar C=C), 917 (Ar C-H). Anal. Calcd for $C_{15}H_{18}N_2O_5$: C, 58.82; H, 5.92; N, 9.15. Found: C, 58.79; H, 5.91; N. 9.17.

Synthesis of P1

Phe NCA (234.6 mg, 1.24 mmol) was dissolved in anhydrous DCM (10 mL). The solution was injected into a flame-dried and nitrogen-purged Schlenk tube. Amine-terminated $mPEG_{113}$, average M_n = 5,000 g/mol (155 mg, 0.031 mmol) was dissolved in anhydrous DCM (10 mL) and the solution was injected into the reaction medium, which was stirred at room temperature, under a nitrogen flow, for 96 hours. The polymer produced was precipitated in cold Et_2O (1:5 v/v), centrifuged and dried *in vacuo*. Yield: 44.6%. ^{1}H NMR (500 MHz, TFA-d): δ 8.35 (s, αCHCONH)), 7.10 - 6.88 (m, Ph), 4.02 (s, αCHCONH), 4.02 - 3.31 (m, $mPEG_{113}$), 2.96 - 2.81 (m, Ar-CH_2). FTIR: ν_{max}/cm^{-1} (solid): 3287 (NH), 2882 (C-H), 1632 (CO amide), 1537 (NH), 1342 (Ar C=C), 1094 (PEG), 697 - 958 (Ar C-H).

Synthesis of Cbz-protected P2-P4

A representative procedure is given for a target monomer-initiator molar feed ratio of 40 (**P3**). Lys(Cbz) NCA (380.2 mg, 1.24 mmol) was dissolved in anhydrous DCM (10 mL). The solution was injected into a flame-dried and nitrogen-purged Schlenk tube. Amine-terminated $mPEG_{113}$, average M_n = 5,000 g/mol (155 mg, 0.031 mmol) was dissolved in anhydrous DCM (10 mL). The solution was injected into the reaction medium. Further steps were carried out as described for the synthesis of $poly(Glu(Bz))_n$. Yield: [M/I] = 20: 88.3%; [M/I] = 40: 96.2%; [M/I] = 75: 94.6%. ^1H NMR (500 MHz, TFA-d): δ 8.49 (s, CONH), 7.43 (m, *J* = 35.8 Hz, Ph (Cbz)), 5.26 (s, Ar-CH_2), 4.67 (s, αCHNH), 4.20 – 3.77 (m, methoxy-PEG_{113}), 3.30 (d, *J* = 12.0 Hz, CH_2NHCOO), 2.16 - 1.75 (m, $αCHCH_2$), 1.75 -1.15 (m, $CH_2CH_2CH_2NH$). FTIR: ν_{max}/cm^{-1} (solid): 3710-3283 (NH, amide & amine), 2892 (C-H), 1652 (C=O amide), 1537 (NH), 1343 (C-H), 1114 (PEG), 1774 (Bz ester), 1686 (Ar C=C), 917 (Ar C-H).

Deprotection of Cbz-protected P2-P4

Protected polymer (480 mg) was dissolved in TFA (7 mL) and added to a 33 wt.% solution of HBr in TFA (3 mL). The mixture was stirred at room temperature for 24 hours, then added dropwise into cold Et_2O (150 mL) to induce the precipitation of the deprotected polymer. The precipitate was isolated by centrifugation (3000 rpm, 10 min). The polymer was re-suspended in fresh Et_2O, stirred for 30 min and centrifuged. This was repeated several times until a clear supernatant was obtained. The polymer was dissolved in de-ionised H_2O and dialysed against de-ionised water for 96 hours, with the dialysate being substituted with a fresh supply after every 8 hours. The polymer was isolated by freeze-drying.

	Yield (mg)	m:n*
**P2**	171	1:20
**P3**	370	1:40
**P4**	548	1:75

*Estimated from ^{1}H NMR spectra by normalising to $mPEG_{113}$ integrals and comparing the mPEG signal (3.40 ppm - 3.52 ppm) to the proton signal from α-CH groups of poly(L-lysine) (3.25 ppm).

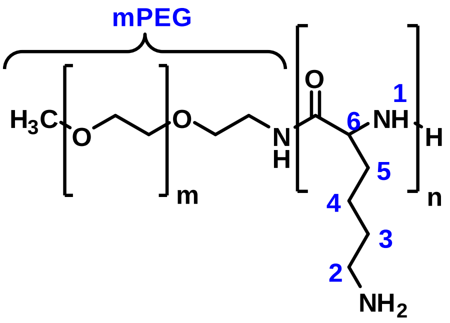

^{1}H NMR (300 MHz, DMSO-d_6): δ 8.03 (broad s, N*H*, **1**), 4.27 (broad s, C*H*, **6**), 3.51 (broad s, mPEG), 2.78 (broad s, CH_2, **2**), 1.66-1.57 (broad m, CH_2, **3/4**), 1.35 (broad s, CH_2, **5**).

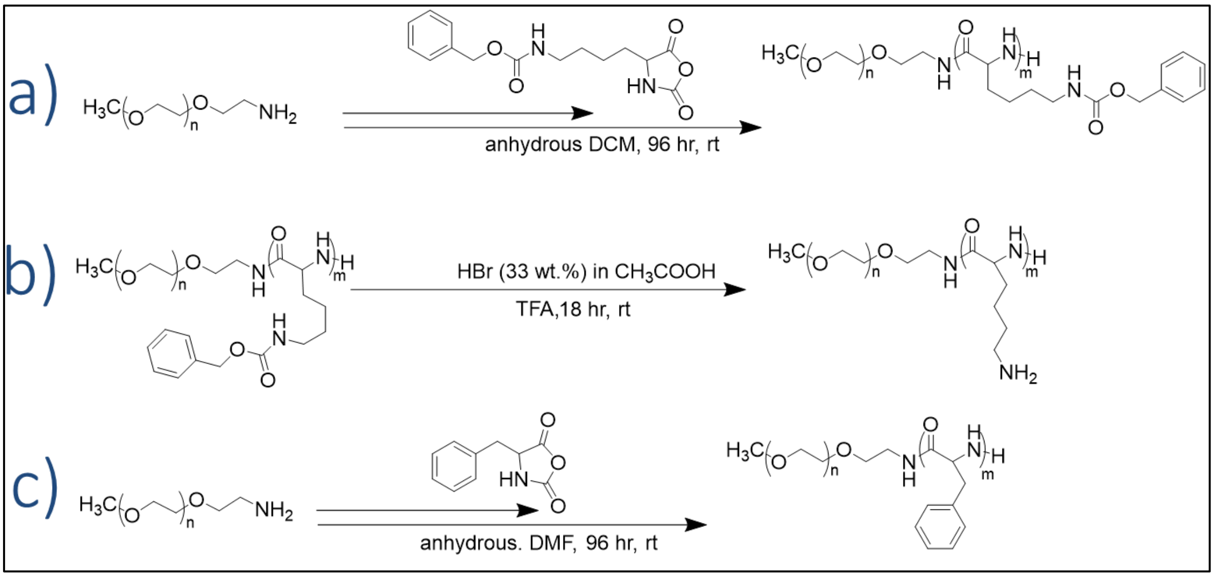

a) Syntheses of mPEG-b-Poly(Lys(Cbz)) (**P2, P3, P4**); **b)** Poly(amino acid) deprotection of **P2, P3, P4**; **c)** Synthesis of mPEG-poly(Phe) (**P1**).

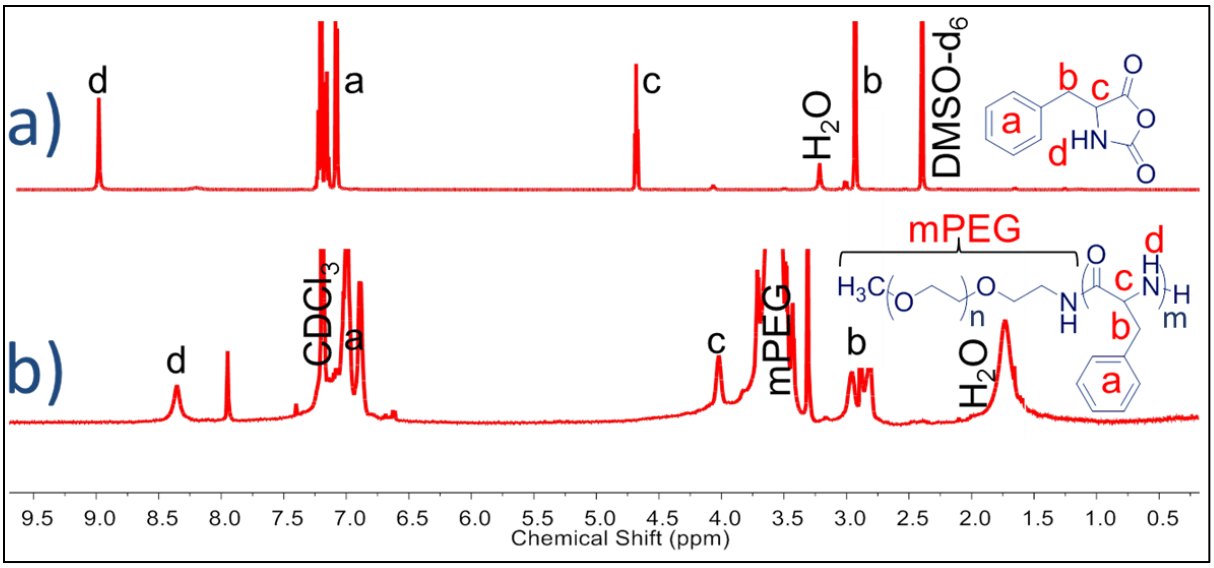

^{1}H NMR spectra of **a)** L-phe NCA; **b)** mPEG-b-poly(L-phe) (**P1**).

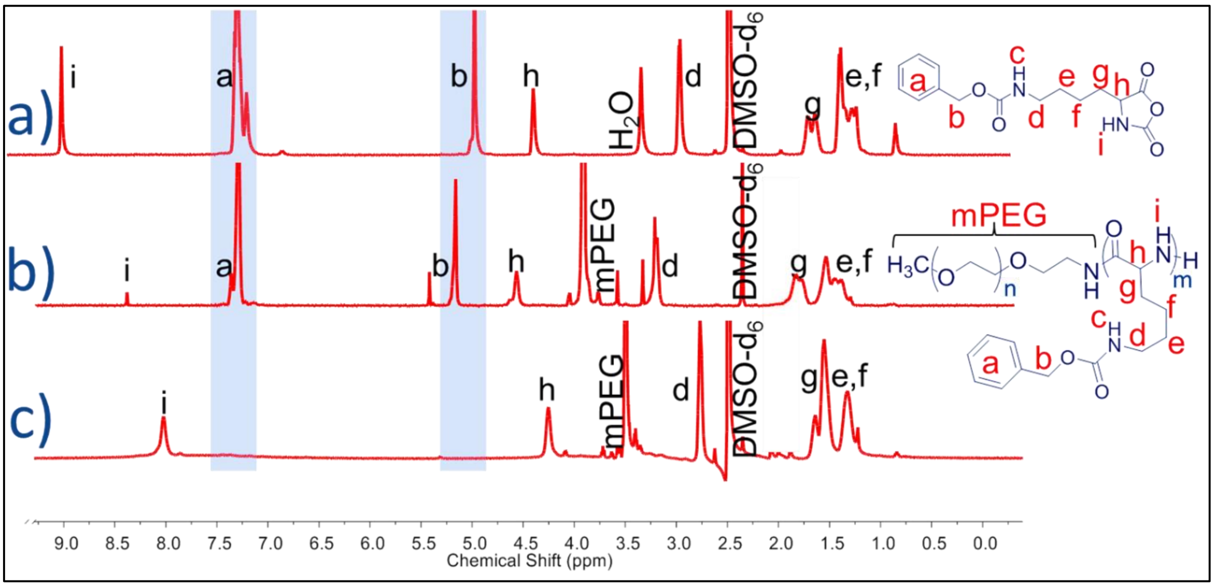

^{1}H NMR spectra of **a)** Lys(Cbz) NCA; **b)** mPEG-b-poly(Lys(Cbz)) (representative of Cbz-protected **P2, P3, P4**); **c)** mPEG-b-poly(Lys) (representative of poly(amino acid) deprotected **P2, P3, P4**).

Self-Assembly of P1 in the presence of C1

Stock solutions of **P1** (1 mg/mL) in DMF and **C1** (0.25 mg/mL) in H_2O were prepared by serial dilution. 20, 40, 200 and 400 µL of the **P1** solution were added to 2 mL of the **C1** solution. The solutions were shaken on a vibrax machine, and maintained

under dialysis to determine any non-uptake of inorganic matter. The average particle size and polydispersity index (PDI) values were measured using dynamic light scattering (DLS).

	**P1** = 20µL (0.02mg)	**P1** = 40µL (0.04mg)	**P1** = 200µL (0.2mg)	**P1** = 400 µL (0.4mg)
**C1** = 2000 µL (0.5mg)	Particle size: 301.7 nm<br>PDI: 0.884	Particle size: 277.4 nm<br>PDI: 0.983	Particle size: 260.1 nm<br>PDI: 0.984	Particle size: 151.7 nm<br>PDI: 0.307

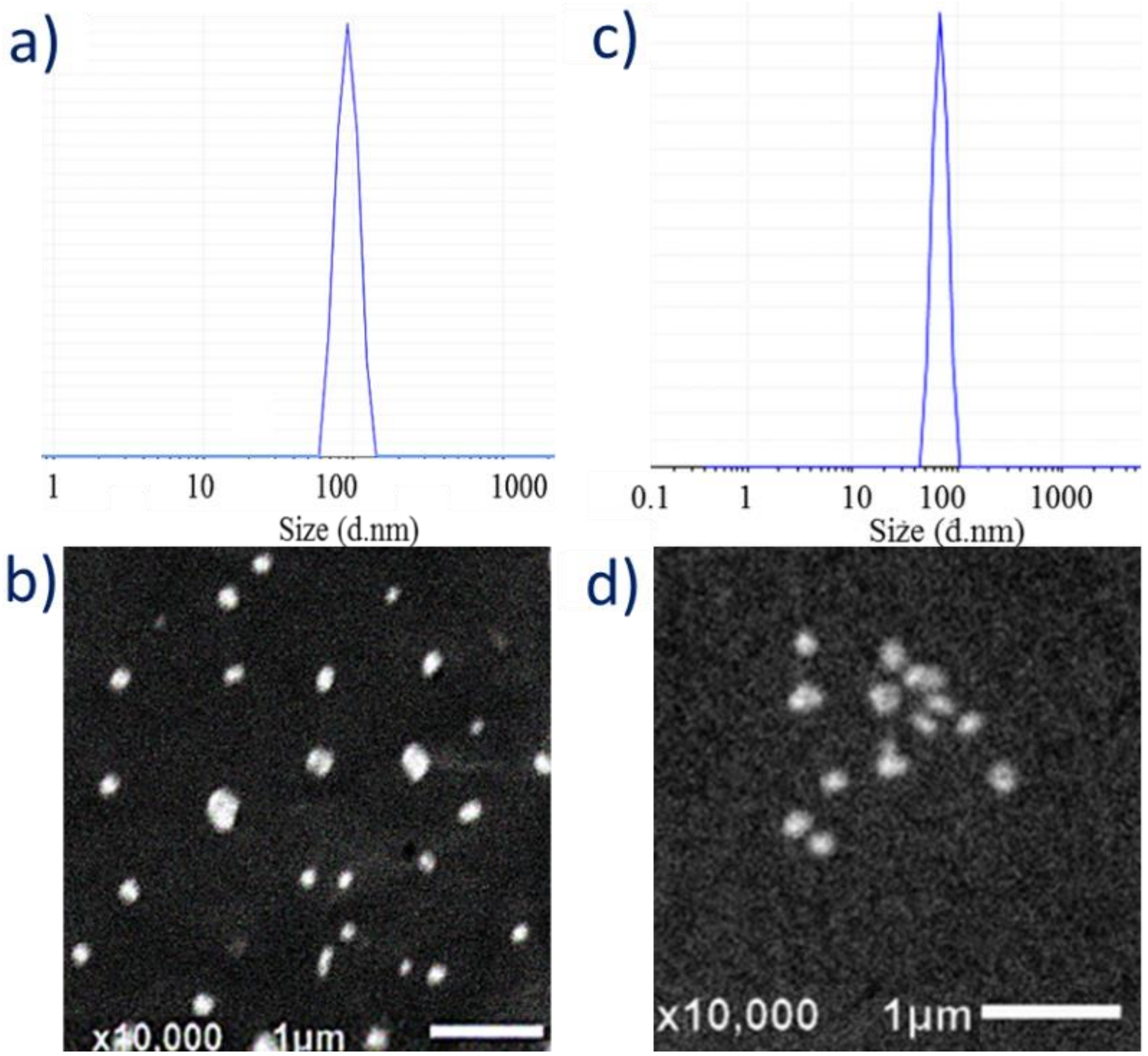

DLS charts revealing the size distribution of **a) P1:C1**; **c) P1:C2**. SEM microphotographs of **b) P1:C1**; **d) P1:C2**. Scale bars represent 1 µm.

Creation of nanoparticles by silver-NHC complex-mediated polymer self-assembly

Solutions (1 mg/mL) of **P2**, **P3**, **P4**, **L3**, **L4**, **C3** and **C4** were prepared in HPLC grade water, and filtered through 0.45 µm and 0.2 µm filters and analysed using DLS to confirm no particles were present in the absence of polymer-silver-NHC complex interaction. Equal volumes (1 mL) of each polymer solution and either ligand or complex were mixed to form solutions of 1:1 ratio, and agitated using a vibrax machine. The particles in solution were maintained under dialysis to determine any non-uptake of inorganic matter, heated to 37 °C and analysed by DLS (Table 1 in manuscript).

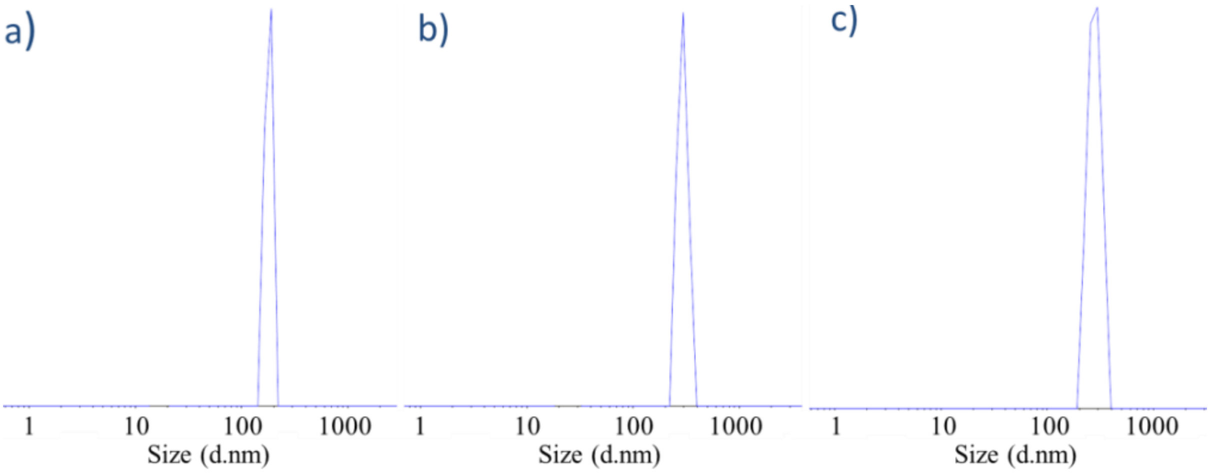

DLS charts obtained from **a) P2**:**C3**; **b) P3**:**C3**; **c) P4**:**C3**.

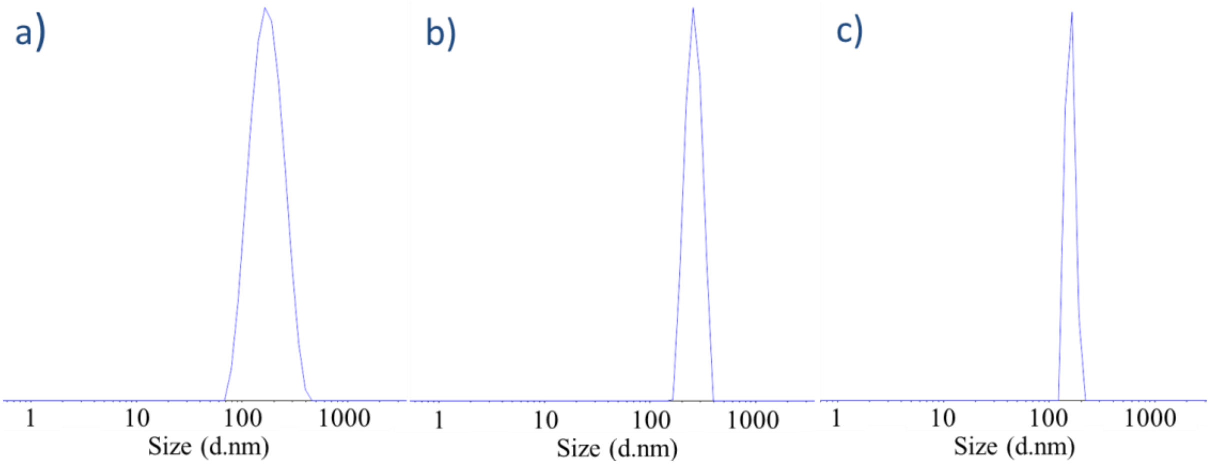

DLS charts obtained from **a) P2:C4**; **b) P3:C4**; **c) P4:C4**.

Cell line testing

In vitro cell tests were performed at the Department of Pharmacy, University of Huddersfield. Cells were incubated in 96-well plates, at 2 x 10^3 cells per well in 200 µL of growth media (RPMI 1640 supplemented with 15 % foetal calf serum, 10 units/mL human recombinant insulin, sodium pyruvate (1 mM) and L-glutamine (2 mM) for Panc 10.05 cell line; DMEM:F12 10 % foetal calf serum, sodium pyruvate (1mM) and L-glutamine (2 mM) for ARPE-19 cell line). Cells were incubated for 24 hours at 37 ºC in an atmosphere of 5 % CO_2 prior to drug exposure. All compounds were dissolved in DMSO at a concentration of 100 mM and diluted with medium to obtain drug solutions ranging from 100 µM to 0.049 µM. The final DMSO concentration was 0.1 % (v/v) which is non-toxic to cells. Drug solutions were applied to cells and incubated for 96 hours at 37 ºC in an atmosphere of 5 % CO_2. The solutions were removed from the wells and fresh medium added to each well along with 20 µL MTT (5 mg/mL), and incubated for 4 hours at 37 ºC in an atmosphere of 5 % CO_2. The solutions were removed and 150 µL DMSO was added to each well to dissolve the purple formazan crystals. A plate reader was used to measure the absorbance at 540 nm. Lanes containing medium only, and cells in medium only (no drug), were used as blanks for the spectrophotometer and 100 % cell survival respectively. Cell survival was determined as the absorbance of treated cells divided by the absorbance of controls and expressed as a percentage. The concentration required to kill 50 % of cells (IC_{50}) was determined from plots of percent survival against drug concentration. Each experiment was repeated 3 times and a mean value obtained.

For studies using polymer complexes, the polymer was dissolved in distilled water at 1 mg/mL and vortexed to ensure dispersion. Similarly, the test compound was dissolved in distilled water at 1 mg/mL and a 50:50 mix of polymer and test compound prepared. The mix was then vortexed to ensure adequate mixing and dispersion. Samples were diluted in cell culture media to provide a concentration of test compound at 100 µM (the polymer concentration was less than 0.01 % w/v in all cases). A range of test drug concentrations were prepared by diluting the highest concentration with media containing polymer (at less than 0.01 % w/v) so the concentration of polymer remained constant across all lanes. The control contained

media plus polymer only (no test drug included) and chemosensitivity studies were performed as described above.
